# Ghrelin Receptor Is Required for the Effect of Nesfatin-1 on Glucose Metabolism

**DOI:** 10.3389/fendo.2018.00633

**Published:** 2018-10-24

**Authors:** Xin-Tong Fan, Zhao Tian, Shi-Zhen Li, Ting Zhai, Jun-Li Liu, Rui Wang, Cai-Shun Zhang, Liu-Xin Wang, Jun-Hua Yuan, Yu Zhou, Jing Dong

**Affiliations:** ^1^Clinical Medicine Department, Medical College, Qingdao University, Qingdao, China; ^2^Preventive Medicine Department, School of Public Health, Qingdao University, Qingdao, China; ^3^Fraser Laboratories for Diabetes Research, Department of Medicine, The Research Institute of McGill University Health Centre, Montreal, QC, Canada; ^4^Special Medicine Department, Medical College, Qingdao University, Qingdao, China; ^5^Physiology Department, Medical College, Qingdao University, Qingdao, China

**Keywords:** nesfatin-1, ghrelin receptor, glucose metabolism, AKT, high fat diet

## Abstract

Studies of nesfatin-1 in glucose metabolism have become a topic of interest recently, however, the specific receptor for nesfatin-1 has not yet been identified. Some studies hinted at a connection between nesfatin-1 and the ghrelin receptor, growth hormone secretagogue receptor. Therefore, we aimed to study the role of GHSR in the glycemic effects of nesfatin-1 as well as its downstream pathways. We employed C57/BL6 mice (wild type and GHSR knockout mice) eating a normal chow diet and a high fat diet in this study, and the experimental technique included western blot, real-time PCR, immunofluorescence and ELISA. We found that in mice fed a normal chow diet (NCD), nesfatin-1 improved glucose tolerance, up-regulated AKT kinase (AKT) mRNA levels and phosphorylation and GLUT4 membrane translocation in skeletal muscle. These effects were blocked by co-injection of GHSR antagonist [D-Lys3]-GHRP-6 and were attenuated in GHSR knockout mice. In mice fed high-fat diet (HFD), nesfatin-1 not only exerted the effects observed in NCD mice, but also suppressed appetite and raised AKT levels in liver tissues that also required GHSR. Peripheral nesfatin-1 suppressed c-fos expression of GHSR immunoreactive neurons induced by fasting in hypothalamic nuclei, indicating that nesfatin-1 inhibited the activation of central GHSR. We concluded that the effects of nesfatin-1 on food intake and glucose metabolism were GHSR-dependent, and that the glycemic effect was associated with AKT and GLUT4. This study should stimulate further exploration of the nesfatin-1 receptor.

## Introduction

Nesfatin-1 is an anorexic peptide discovered in 2006 (1). Protein nucleobindin-2 (NUCB2), its precursor, was found to be related to severe obesity in human children (2). Nesfatin-1 immunoreactive cells are distributed in central neurons (3–5) and some peripheral tissues, including gastric mucosa, heart, islet cells, and adipose tissue (6–10). It has been shown to inhibit food intake and reduce body weight both centrally (1, 11) and peripherally (12), suggesting a potential therapeutic role in obesity.

Nesfatin-1 also participates in regulation of blood glucose. Continuous subcutaneous administration of nesfatin-1 diminishes blood glucose levels during the oral glucose tolerance test (OGTT), while intracerebroventricular (ICV) injection of nesfatin-1 did not reduce blood glucose levels (13), suggesting that the glycemic effect of nesfatin-1 may be peripheral. Another study found that the effect of peripheral nesfatin-1 on reducing blood glucose was mediated in a glucose- and insulin-dependent manner (14). In addition, nesfatin-1 decreased blood glucose indirectly in other organs: co-injection of insulin and nesfatin-1 increased phosphorylation levels of AKT kinase (AKT) in liver, skeletal muscle, and adipose tissue, and thus improved GLUT4 levels to increase glucose uptake (13).

Ghrelin, purified from rat stomach in 1999 by Kojima et al. (15), has also emerged as a pivotal player in the regulation of appetite and energy metabolism. Both central and peripheral injection of ghrelin increased appetite and body weight (16, 17). Studies have found that ghrelin promoted the synthesis of liver glycogen (18), increased blood glucose, and inhibited insulin release (19), all of which underscore the important effect of ghrelin in modulating glucose metabolism. Growth hormone secretagogue receptor (GHSR), considered the main receptor for ghrelin (20), was found to be expressed in pancreatic β-cells (21–23), and insulin levels decreased in GHSR knockout mice (24). This suggested a critical role for GHSR in regulating glucose homeostasis. More importantly, a study illustrated that GHSR had high constitutive activity independent of ghrelin (25). This implied that ligand-independent signaling can be affected by other unidentified inverse agonist, to exert the corresponding functions.

Together, nesfatin-1 and ghrelin both play a profound part in regulating the energy state. Interestingly, nesfatin-1 and ghrelin were both secreted by X/A-like cells and co-localized in gut and brain (26, 27). Kerbel et al. employed goldfish to study the relationship between the two brain-gut peptides and found that nesfatin-1 injection changed the expression of ghrelin and its active receptor GHSR 1a-1 (26). Another study suggested that nesfatin-1 interacted with the G-protein-coupled receptors in mouse brain (3). Interestingly, GHSR was also shown to be a G protein-coupled receptor (28). Furthermore, nesfatin-1 and GHSR co-exist in human pancreatic beta cells (29), while there is no ghrelin in human beta cells (30). In addition, GHSR and nesfatin-1 in beta cells were closely related to the regulation of insulin secretion (31–33). Based on all these observations, because the nesfatin-1 receptor has not been identified, we hypothesized that nesfatin-1 may have a direct or indirect effect on GHSR when modulating glucose metabolism. Based on the results we obtained, we confirmed our hypothesis that nesfatin-1 modulated satiety, and that glucose metabolism mediated by GHSR at least partly requires its participation.

## Materials and methods

### Animals

Adult male wild-type (GHSR^+/+^) and GHSR-null (GHSR^−/−^) mice (age 8 weeks) (34) on a C57BL/6J background, were housed in standard rodent cage. The original GHSR^−/−^ mice were purchased from Shanghai Research Center For Model Organisms Of China, and the GHSR^−/−^ mice were obtained from crosses between heterozygous and homozygous knockout animals that were backcrossed over 10 generations (35). The environment conditions were controlled (23 ± 2°C, illumination from 0700 to 1900 h). Food and water were available *ad libitum*, except during the specified experiments. The experimental protocols were approved by the Qingdao University Animal Care and Use Committee and Animal Welfare Committee in accordance with the National Institutes of Health guidelines.

### Experimental procedures

Eight-week-old mice were allowed free access to standard laboratory normal chow diet (NCD, 10 % calories from fat: 51.5% wheat, 20% fat-free milk, 11.25% soybean meal, 10.25% vegetable oil, 4% bear yeast, 1.375% salt, 0.125% ferric citrate, 0.5% vitamins, 1% calcium hydrophosphate. Qingdao Daren Fortune Animal Technology) or high-fat diet (HFD, 60 % calories from fat: 59% basic mice feed, 20% sugar, 18% lard, and 3% egg yolk) for 8 weeks. Before the experimental procedures, mice were housed individually and habituated to the irritation of intravenous administration.

#### Experimental groups

A. GHSR^+/+^ mice fed NCD were randomly divided into four groups according to a random number table. Vehicle (normal saline, *n* = 6) or nesfatin-1 (100 pmol/mouse/day, 1–82; Phoenix Pharmaceuticals, Burlingame, CA, USA, *n* = 6) or [D-Lys3]-GHRP-6 (GHSR antagonist, 16.7 μg/mouse/day; ApexBio, Houston, USA, *n* = 6) or co-injection of nesfatin-1 and [D-Lys3]-GHRP-6 (*n* = 8) was injected into the tail vein daily for 12 days (13). Nesfatin-1 and [D-Lys3]-GHRP-6 were premixed into one solution before the co-injection of the two drugs. B. GHSR^+/+^ mice fed HFD were randomly divided into two groups. Vehicle (*n* = 6) or nesfatin-1 (*n* = 6) was injected into the tail vein daily for 12 days. C. GHSR^−/−^ mice fed NCD and HFD were randomly divided into two groups (vehicle and nesfatin-1), respectively (NCD, vehicle: *n* = 3; NCD, nesfatin-1: *n* = 4; HFD, vehicle: *n* = 5; HFD, nesfatin-1: *n* = 5). Drugs were injected daily for 12 days.

#### Experimental procedures

Administrations were conducted in the evening at 1800 h before the onset of the dark cycle (day 1-11) in mice fasting for 6 h (except OGTT), with the last injection at 0900 h in the morning (day 12) after mice fasted for 12 h. When indicated, wild-type mice and GHSR knockout mice underwent treatment with intraperitoneal insulin (2 U/kg body weight; Wan Bang, China) 10 min before sacrifice, since the effect of peripheral nesfatin-1 on regulating AKT and GLUT4 was insulin-dependent (13). Mice were sacrificed 1.5 h after the last administration of vehicle, nesfatin-1, GHSR antagonist or the co-injection of nesfatin-1 and GHSR antagonist. Skeletal muscle and liver tissue were harvested, temporarily placed in liquid nitrogen, and promptly transferred to a −80°C freezer (Thermo Scientific ^TM^) until they were assayed for AKT phosphorylation (p-AKT/AKT), GLUT4 protein expression levels, and mRNA levels of AKT and GLUT4. The inclusion and exclusion of data were based on the accuracy of experimental procedures and surgeries. All experimenters were blind to group assignment and outcome assessment. To reduce experimental error, we conducted several measurements for each sample and every substantial deviation from normal value was repeated to improve the accuracy of the experiment.

### Food intake

Food intake was detected during the 12-day administration mentioned above. Food was returned to the mice immediately after tail vein administration and was measured by electronic precision scales (Feeding and Activity Analyzer, Ugo Basile, Italy) at 0.5, 1, 2, 3, and 12 h after injection.

### Body weight and glucose measurement

Body weight (initial weights: 18–22 gram) was measured on a weekly basis during high fat diet (HFD) with an electric balance (Mettler Toledo, PL1501-S, Shanghai, China). For the effect of nesfatin-1 modulating acute blood glucose (BG), we measured BG using tail vein prick and a glucometer (B, Braun, Meisungen AG, Germany) before administration, and at 1 and 3 h after the injection during the 12-day administration period. For the effect of nesfatin-1 regulating chronic BG, we measured BG at the beginning and the end of the 12-day administration (before mice were sacrificed).

### Oral glucose tolerance test (OGTT)

OGTT was performed on day 8 during the 12-day administration period. The mice were fasted for 16 h before OGTT (36) and were then injected with vehicle or nesfatin-1, after which they were immediately treated with glucose (3 g/kg body weight) orally. Blood glucose concentration was measured at 0, 15, 30, 60, 120 min after gavage, using the glucometer described above.

### Biochemical analysis

Blood was sampled from the tip of the tails of GHSR^+/+^ and GHSR^−/−^ mice that had been fed *ad libitum* or fasted for 12 h. Plasma was immediately transferred into EP tubes to determine the levels of nesfatin-1. Plasma nesfatin-1 was measured by ELISA (Cloud-Clone Crop, USA, CEA242Mu).

### Real-time PCR

TRIzol reagent (Vazyme Biotech Co., Ltd, China) was used to isolate total RNA from liver and skeletal muscle. By measuring absorbance at 260 nm, we quantified the RNA concentration and used the ratio of absorbance at 260 and 280 nm in a spectrophotometer to assess the purity of RNA. RNA was then reverse transcribed into cDNA using HiScript II Q RT SuperMix for qPCR (Vazyme Biotech Co., Ltd, China). The obtained cDNA was used to determine the mRNA levels of AKT and GLUT4 (skeletal muscle, liver) by real-time PCR using ChamQ SYBR qPCR Master Mix (Vazyme Biotech Co., Ltd, China). The primer sequences (designed by Shanghai Sangon, China) are shown in Table 1. Amplification was initiated at 95°C for 10 min, followed by 35 cycles at 95°C for 10 s, and then at 60°C for 30 s and finally terminated at 72°C for 30 s. The relative expression levels of AKT and GLUT4 were normalized to β-actin threshold cycle (Ct) values, and the fold changes of each target gene were calculated using the 2(-ΔΔCt) method.

**Table 1:** Premier sequences for real-time PCR.

**Gene**	**Forward**	**Reverse**
AKT serine/threonine kinase 1 (AKT1)	5′-AGATTGTGTCGCCCGGAC-3′	5′-AGCCCGAAGTCCGTTATCTT-3′
Solute carrier family 2 member 4 (GLUT4)	5′-TCTTATTGCAGCGCCTGAGTC-3′	5′-GCCAAGCACAGCTGAGAATACA-3′
Actin beta (β-actin)	5′-CCACTGCCGCATCCTCTTCC-3′	5′-CTCGTTGCCAATAGTGATGAC-3′

### Western blot

We extracted proteins from liver, skeletal muscle with RIPA Lysis Buffer (Beyotime, P0013B, China). Proteins were denatured in SDS sample buffer (95°C, 5 min) and then were subjected to 12% SDS-PAGE.

Proteins were then transferred on a polyvinylidene fluoride membranes (Millipore Corp., Billerica, MA, USA) for 2 h. Blocking was performed, and membranes were incubated overnight (4°C) in primary antibodies (p-AKT: rabbit IgG, 1:2000, Cell Signaling Technology, PSER473, #4060, USA; AKT: rabbit IgG, 1:2,000, Sigma, SAB4500797, Germany; GLUT4: rabbit IgG, 1:2,000, Abcam, ab33780, US; β-actin: rabbit IgG, 1:2,000, Cell Signaling Technology, D6A8, #8457, USA). Secondary antibodies (Goat Anti-rabbit IgG, 1:8,000, Boaosen, bs-0295G-HRP, China) were incubated on the membranes for 1 h at room temperature. Detection of proteins was performed with an enhanced chemiluminescence reagent (Millipore, WBKLS0100, USA). Image J was used for intensity analysis.

### Immunofluorescence

All mice used for immunofluorescence were fasted for 12 h before IV treatment (37). C57/BL6 mice had access to free time for 90 min after tail vein injection with nesfatin-1(100 pmol/mouse, 1–82; Phoenix Pharmaceuticals, Burlingame, CA, USA, *n* = 3) or vehicle (*n* = 3), and after this period, the mice were deeply anesthetized with chloral hydrate, then quickly perfused transcardially with 30 ml 0.1M PBS, followed by 30 ml 4% paraformaldehyde in 0.1 M PBS. The brains were quickly removed immediately following perfusion and were post-fixed in 4% paraformaldehyde overnight at 4°C. After post-fixation, the brains were transferred to 20% sucrose solutions for 12 h, then were moved to 30% sucrose solution for another 12 h. The brains were sectioned at 15 μm on a freezing microtome (Kryostat 1,720; Leica, Germany), and sections were placed on glass slides. Then sections were soaked in 0.01M PBS for 15 min, followed by antigen retrieval in a microwave oven. Then sections were washed in 0.01M PBS three times (7 min per wash). After washing, sections were blocked for 2 h at room temperature in 0.01 M PBS buffer with 1% BSA containing 0.1% Triton-X, then incubated overnight at 4°C in primary antibody against c-fos (sheep anti-c-fos polyclonal antibody, 1:200, Millipore) diluted in blocking solution. Following three washes in 0.01M PBS, sections were incubated with secondary antibody (donkey anti-sheep, red, 1:200, R&D) diluted in PBS for 2 h at room temperature. Then they were mounted with coverslips with 50% glycerol after three PBS washes.

For the evaluation of GLUT4 membrane translocation, muscle tissues were harvested 90 min after tail vein injection with nesfatin-1 (100 pmol/mouse) or vehicle in GHSR^+/+^ and GHSR^−/−^ mice fasted for 12 h, with intraperitoneal insulin (2 U/kg body weight) 10 min before sacrifice (*n* = 3/group). After perfusion described above, muscle was post-fixed in 70, 80, 90, 100, 100% paraformaldehyde for 30 min, respectively. Then muscle was transferred into dimethylbenzene for 4 min, following immersion in soft wax, hard wax, and mixed waxes for 40 min, respectively. After sectioning, sections were placed in 100, 100, 90, 80, 70% alcohol solution for 5 min, respectively, followed by dimethylbenzene dewaxing for 20 min. After washing in 0.01M PBS, the following immunostaining protocols were as described above. Blocking buffer: 1% BSA in 0.01M PBS. Primary antibody: Rabbit Anti-Glucose Transporter GLUT4 polyclonal antibody (1:100, ab654, Abcam), incubated overnight at 4°C; Second antibody: Fluorescein-Conjugated AffiniPure Goat Anti-Rabbit IgG (H+L) (1:300, ZSGB-BIO), incubated for 2 h at room temperature.

A confocal scanning laser microscope (FV500, Olympus) was used to visualize the immunofluorescent staining. Image J was used to immunofluorescence analysis.

### Statistical analysis

Data were expressed using means ± standard error of the means (SEMs). We applied student's *t*-test to analyze statistical differences between two groups, and one-way ANOVA for multiple groups. In all cases, *P* < 0.05 was considered significant. GraphPad Prism and SPSS (Statistical Product and Service Solutions) were used for creation of graphs and statistical analyses, respectively.

## Results

### The differences between GHSR^+/+^ and GHSR^−/−^ mice in food intake and blood glucose

**Figure 1:**
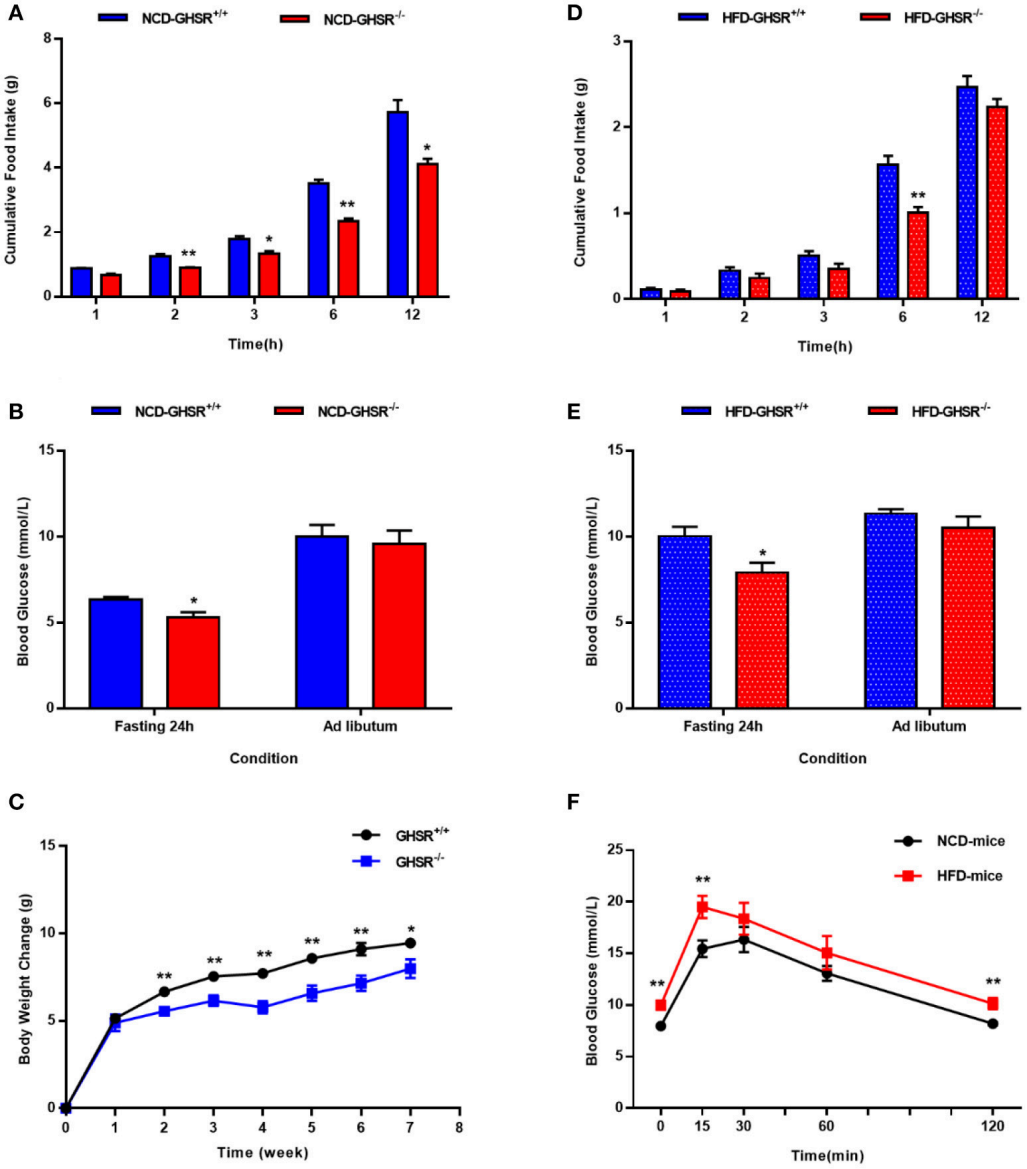
The differences between GHSR^+/+^ and GHSR^−/−^ mice in food intake and blood glucose. Eight-week-old GHSR^+/+^ and GHSR^−/−^ mice were fed with NCD or HFD for 8 weeks: then mice were inspected respectively. **(A,D)** Cumulative food intake in GHSR^+/+^ and GHSR^−/−^ mice fed with **(A)** NCD or HFD tested after mice fasting for 6 h. **(B,E)** Blood glucose in GHSR^+/+^ and GHSR^−/−^ mice fed with **(B)** NCD or **(E)** HFD under *ad libitum* or 24 h fasting conditions. **(C)** Changes in body weight during 8 weeks of HFD. **(F)** Oral glucose tolerance tests (OGTT) in mice fed with NCD and HFD. **(A,B,D,E)** NCD-GHSR^+/+^: *n* = 4; NCD-GHSR^−/−^: *n* = 3; HFD-GHSR^+/+^: *n* = 6; HFD-GHSR^−/−^: *n* = 6. **(C)** *n* = 10/group. **(F)** *n* = 12/group. Data are expressed as mean ± SEM. **(A–E)** ^*^P < 0.05, ^**^P < 0.01 for the effect of GHSR^−/−^ mice vs. GHSR^+/+^ mice; **(F)** ^**^P < 0.01 for the effect of HFD-mice vs. NCD-mice. Student's *t*-test was applied to analyze the statistical difference.

First, we compared food intake and blood glucose state between GHSR^+/+^ and GHSR^−/−^ mice. In accordance with previous reports (38), the deletion of GHSR attenuated cumulative food intake both in mice fed NCD (Figure 1A) and HFD (Figure 1D). Similarly, consistent with the findings of a prior study (35), the difference of *ad libitum* blood glucose levels between GHSR^+/+^ and GHSR^−/−^ mice were not comparable under neither NCD (Figure 1B) or HFD (Figure 1E). However, what we found new was that 24 h-fasted GHSR^−/−^ mice exhibited diminished blood glucose under both NCD and HFD condition. We also found GHSR^−/−^ weighed less than GHSR^+/+^ mice during 8-week breeding (Figure 1C). Additionally, to confirm the effectiveness of HFD, we compared glucose tolerance between NCD and HFD fed mice, and HFD mice showed impaired glucose tolerance as expected (Figure 1F).

### Circulating nesfatin-1 in GHSR^+/+^ and GHSR^−/−^ mice

**Figure 2:**
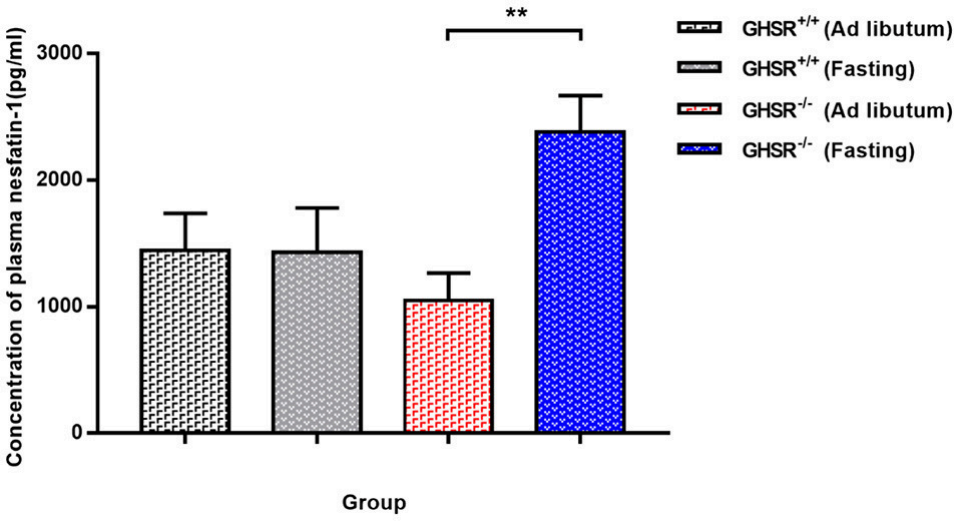
Circulating Nesfatin-1 in GHSR^+/+^ and GHSR^−/−^ Mice. GHSR^+/+^ and GHSR^−/−^ mice were fasted for 12 h or fed *ad libitum*. We obtained blood samples from the end of the tail, after which we performed ELISA. Data are expressed as mean ± SEM (*n* = 6–7/group). ^**^P < 0.01 for the effect of GHSR^−/−^ mice in fasting condition vs. GHSR^−/−^ mice in *ad libitum*. GHSR^−/−^ mice in fasting condition vs. GHSR^+/+^ mice in fasting condition: *P* = 0.05. One-way ANOVA was applied to analyze the statistical difference.

Since a difference was suggested between GHSR^+/+^ and GHSR^−/−^ mice in terms of food intake and blood glucose, and a previous study demonstrated no altered serum leptin and ghrelin levels due to lack of GHSR (39), we measured circulating levels of nesfatin-1 to see whether the difference between these two types of mice correlated with nesfatin-1. In *ad libitum* mice, the differences in concentration of serum nesfatin-1 in GHSR^+/+^ and GHSR^−/−^ mice did not reach statistical significance, but there was a decreasing tendency in GHSR^−/−^ mice (Figure 2). Surprisingly, after 12 h fasting, ELISA showed markedly increased levels of serum nesfatin-1 in GHSR^−/−^ mice compared with levels in mice in the *ad libitum* group, while nesfatin-1 levels in GHSR^+/+^ mice in the two states showed no differences (Figure 2).

### [D-Lys3]-GHRP-6 attenuated nesfatin-1's effects on blood glucose and glucose tolerance

**Figure 3:**
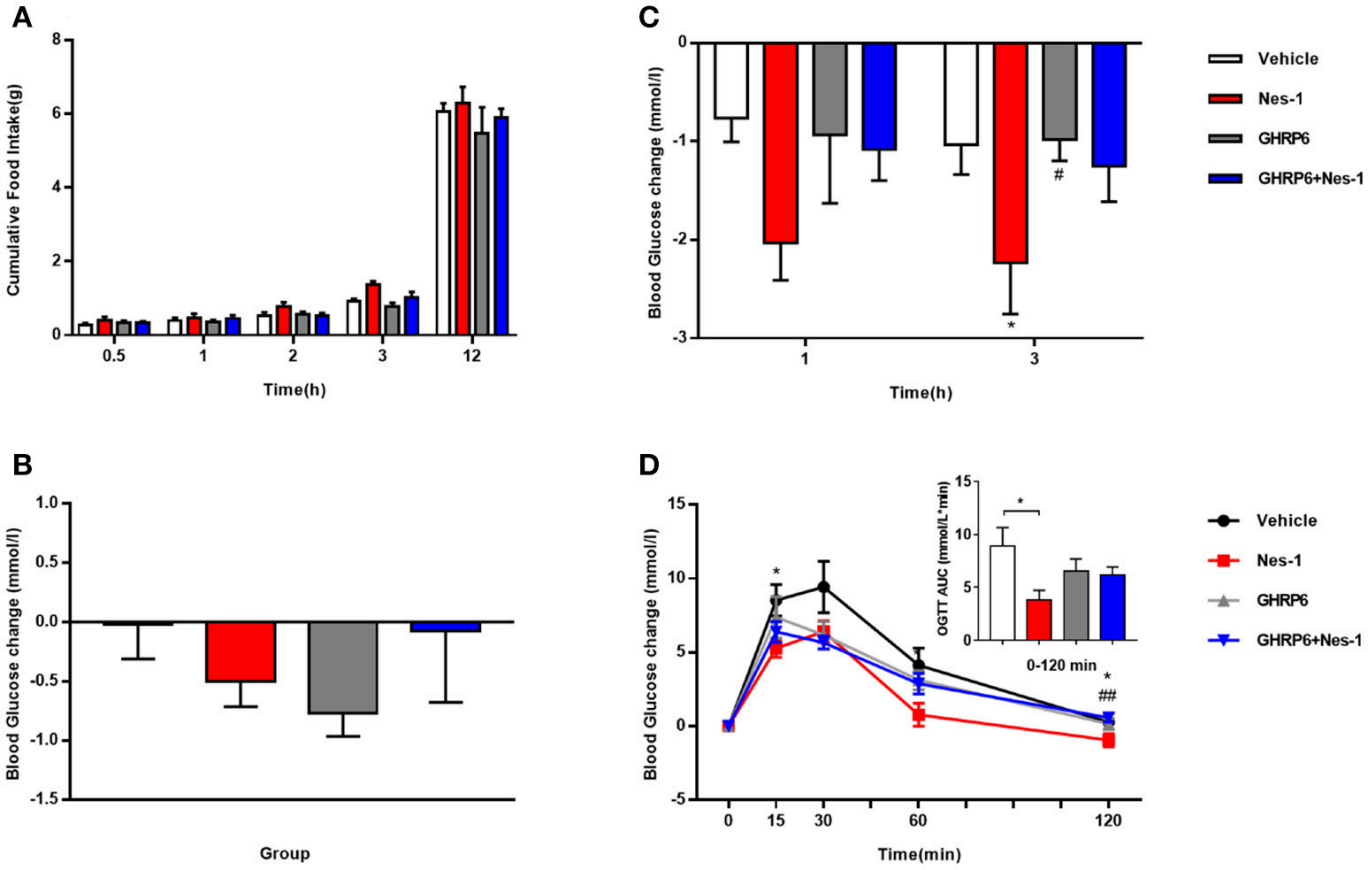
[D-Lys3]-GHRP-6 attenuated nesfatin-1's effects on glucose metabolism. GHSR^+/+^ mice fed NCD underwent chronic tail vein administration of vehicle, nesfatin-1 (Nes-1), [D-Lys3]-GHRP-6 (GHRP-6), and [D-Lys3]-GHRP-6+nesfatin-1 (GHRP-6+Nes-1) for 11 days. **(A)** Food intake and **(C)** blood glucose changes after one acute injection in mice fasting for 6 h. **(B)** Blood glucose changes after 11-day chronic injection compared with drug treatment prior. **(D)** OGTT after 8 days of injection in mice fasting for 16 h. Data are expressed as mean ± SEM. Vehicle: *n* = 6; Nes-1: *n* = 6; GHRP-6: *n* = 6; GHRP-6+Nes-1: *n* = 8. ^*^P < 0.05 for the effect of nesfatin-1 vs. vehicle; ^#^*P* < 0.05, ^##^*P* < 0.01 for the effect of GHRP-6+nesfatin-1 vs. nesfatin-1. One-way ANOVA was applied to analyze the statistical difference.

We employed the GHSR antagonist [D-Lys3]-GHRP-6 to further probe the correlation of GHSR and nesfatin-1 in food intake and blood glucose levels. No significant alteration in cumulative food intake was found between the vehicle and nesfatin-1 groups (Figure 3A). However, we detected significantly lower blood glucose in mice injected with nesfatin-1 after 3 h, and a declining tendency (*P* = 0.056) was observed after 1 h (Figure 3C). Nevertheless, no significant altered blood glucose change was observed in GHSR^+/+^ mice injected with [D-Lys3]-GHRP-6 compared with that of wild-type mice co-injected with nesfatin-1 and [D-Lys3]-GHRP-6 (Figure 3C). We also investigated the role of chronic administration of nesfatin-1 on blood glucose. We monitored blood glucose before and after 11-day chronic administration in four groups of mice (vehicle, nesfatin-1, [D-Lys3]-GHRP-6, and co-injection of nesfatin-1 and [D-Lys3]-GHRP-6). However, no significant difference in blood glucose was observed in mice injected with nesfatin-1 compared with levels in the vehicle group (Figure 3B).

To further investigate the role of GHSR in nesfatin-1's effects on glucose metabolism, we performed OGTT. Nesfatin-1 enhanced glucose tolerance in GHSR^+/+^ mice to a greater extent than in the control group, but blood glucose of mice injected with [D-Lys3]-GHRP-6 was similar to that of mice receiving nesfatin-1 and [D-Lys3]-GHRP-6 co-injection during OGTT (Figure 3D), suggesting the function of nesfatin-1 in improving glucose tolerance was at least partially blocked by GHSR antagonist, which is capable of occupying the GHSR and thus hindering the interaction of nesfatin-1 and GHSR.

### Ghrelin receptor knockout blocked the effects of nesfatin-1 on food intake and glucose metabolism

**Figure 4:**
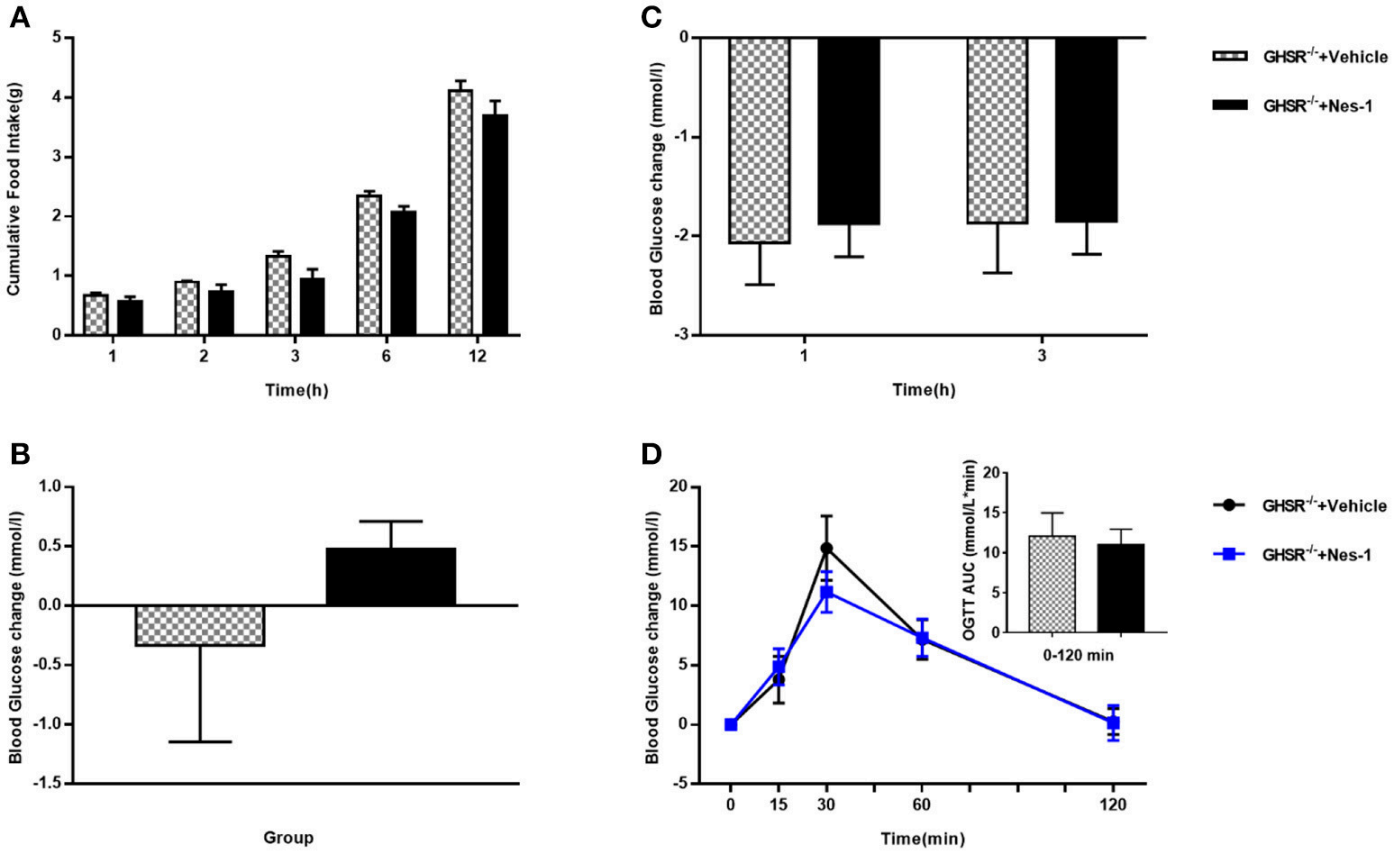
Ghrelin receptor knockout blocked nesfatin-1's effects on glucose metabolism in NCD-fed mice. GHSR^−/−^ mice fed NCD underwent chronic tail vein administration of vehicle or nesfatin-1 (Nes-1) for 11 days. **(A)** Food intake and **(C)** blood glucose changes after one acute injection in mice fasting for 6 h. **(B)** Blood glucose changes after 11-day chronic injection compared with drug treatment prior. **(D)** OGTT after 8 days of injection in mice fasting for 16 h. Data are expressed as mean ± SEM. GHSR^−/−^+Vehicle: *n* = 3; GHSR^−/−^+Nes-1: *n* = 4. No statistical significance is detected. Student's *t*-test was applied to analyze the statistical difference.

**Figure 5:**
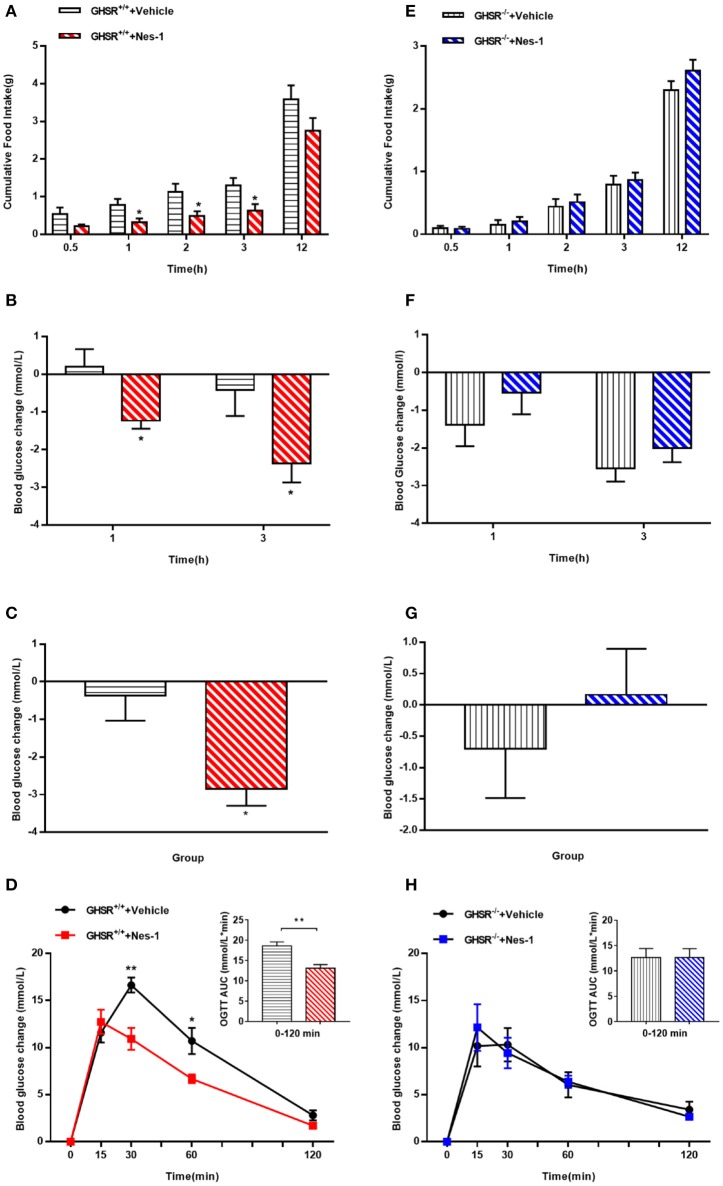
Ghrelin receptor knockout blocked nesfatin-1's effects on food intake and glucose metabolism in HFD-fed mice. GHSR^+/+^ and GHSR^−/−^ mice fed HFD underwent chronic tail vein administration of vehicle or nesfatin-1 (Nes-1) for 11 days, respectively. **(A,E)** Food intake and **(B,F)** blood glucose changes after one acute injection in two types of mice fasting for 6 h. **(C,G)** Blood glucose changes after 11-day chronic injection compared with drug treatment prior. **(D,H)** OGTT after 8 days of injection in mice fasting for 16 h. Data are expressed as mean ± SEM. GHSR^+/+^ +Vehicle, GHSR^+/+^ +Nes-1: *n* = 6/group; GHSR^−/−^ +Vehicle, GHSR^−/−^ +Nes-1: *n* = 5/group. ^*^P < 0.05; ^**^P < 0.01 for the effect of nesfatin-1 vs. vehicle in GHSR^+/+^ mice. Student's *t*-test was applied to analyze the statistical difference.

To move forward another step, GHSR^−/−^ mice were used in the next experiment. We found that IV injection of nesfatin-1 had no impact on cumulative food intake in GHSR^−/−^ mice fed NCD during chronic administration (Figure 4A). Consistent with the prior result, GHSR knockout entirely prevented the effect of nesfatin-1 on glucose metabolism (Figures 4B–D). Here we further employed mice fed with HFD. We found that in HFD-mice, nesfatin-1 distinctly cut down cumulative food intake, acute and chronic blood glucose, and improved glucose tolerance (Figures 5A–D). There was no difference observed between GHSR^−/−^ mice injected with nesfatin-1 and vehicle, just as we observed in NCD-mice (Figures 5E–H).

### GHSR mediated the effect of nesfatin-1 on AKT phosphorylation and GLUT4 membrane translocation

**Figure 6:**
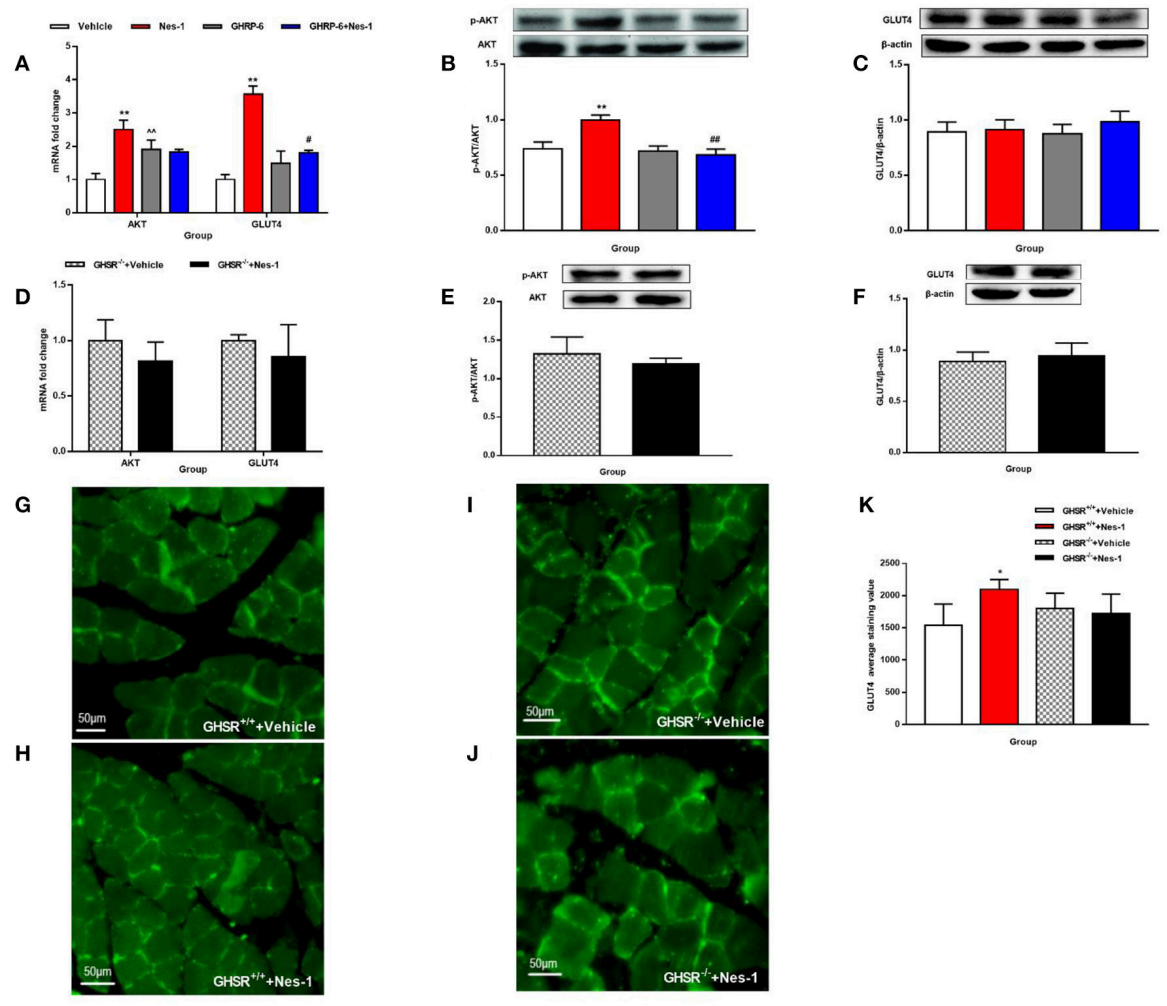
GHSR mediated the effect of nesfatin-1 on AKT phosphorylation and GLUT4 membrane translocation in skeletal muscle. GHSR^+/+^ mice fed NCD underwent chronic tail vein administration of vehicle, nesfatin-1 (Nes-1), [D-Lys3]-GHRP-6 (GHRP-6), and [D-Lys3]-GHRP-6+nesfatin-1 (GHRP-6+Nes-1) for 12 days. GHSR^−/−^ mice fed NCD underwent chronic tail vein administration of vehicle and nesfatin-1 for 12 days. Insulin (2 IU/kg) was intraperitoneally injected into mice 10 min before mice were sacrificed. **(A,D)** mRNA expression levels of AKT and GLUT4 detected by RT-PCR in skeletal muscle of **(A)** GHSR^+/+^ and **(D)** GHSR^−/−^ mice. **(B,E)** P-AKT and **(C,F)** GLUT4 protein levels detected by Western blotting were normalized to total AKT and β-actin, respectively. **(B,E)** P-AKT/AKT level in skeletal muscle of **(B)** GHSR^+/+^ and **(E)** GHSR^−/−^ mice. **(C,F)** GLUT4 level in skeletal muscle of **(C)** GHSR^+/+^ and **(F)** GHSR^−/−^ mice. **(G,H)** GLUT4 membrane translocation detected by immunofluorescence in skeletal muscle of GHSR^+/+^ mice treated with **(G)** vehicle and **(H)** nesfatin-1. **(I,J)** GLUT4 membrane translocation detected by immunofluorescence in skeletal muscle of GHSR^−/−^ mice treated with **(I)** vehicle and **(J)** nesfatin-1. **(K)** GLUT4 average staining value in skeletal muscle of GHSR^+/+^ mice and GHSR^−/−^ mice. *n* = 5/group; **(D)** GHSR^−/−^ + Vehicle: *n* = 3, GHSR^−/−^ + Nes-1: *n* = 4; **(E,F)** *n* = 3/group; **(G–K)** *n* = 3/group. Data are expressed as mean ± SEM. ^*^P < 0.05; ^**^P < 0.01 for the effect of nesfatin-1 vs. vehicle; ^#^*P* < 0.05, ^*##*^*P* < 0.01 for the effect of GHRP-6+nesfatin-1 vs. nesfatin-1; ^∧∧^*P* < 0.01 for the effect of GHRP-6 vs. vehicle. **(A–C,K)** One-way ANOVA was applied to analyze the statistical difference for multiple groups. **(D–F)** Student's *t*-test was applied to analyze the statistical difference for two groups.

**Figure 7:**
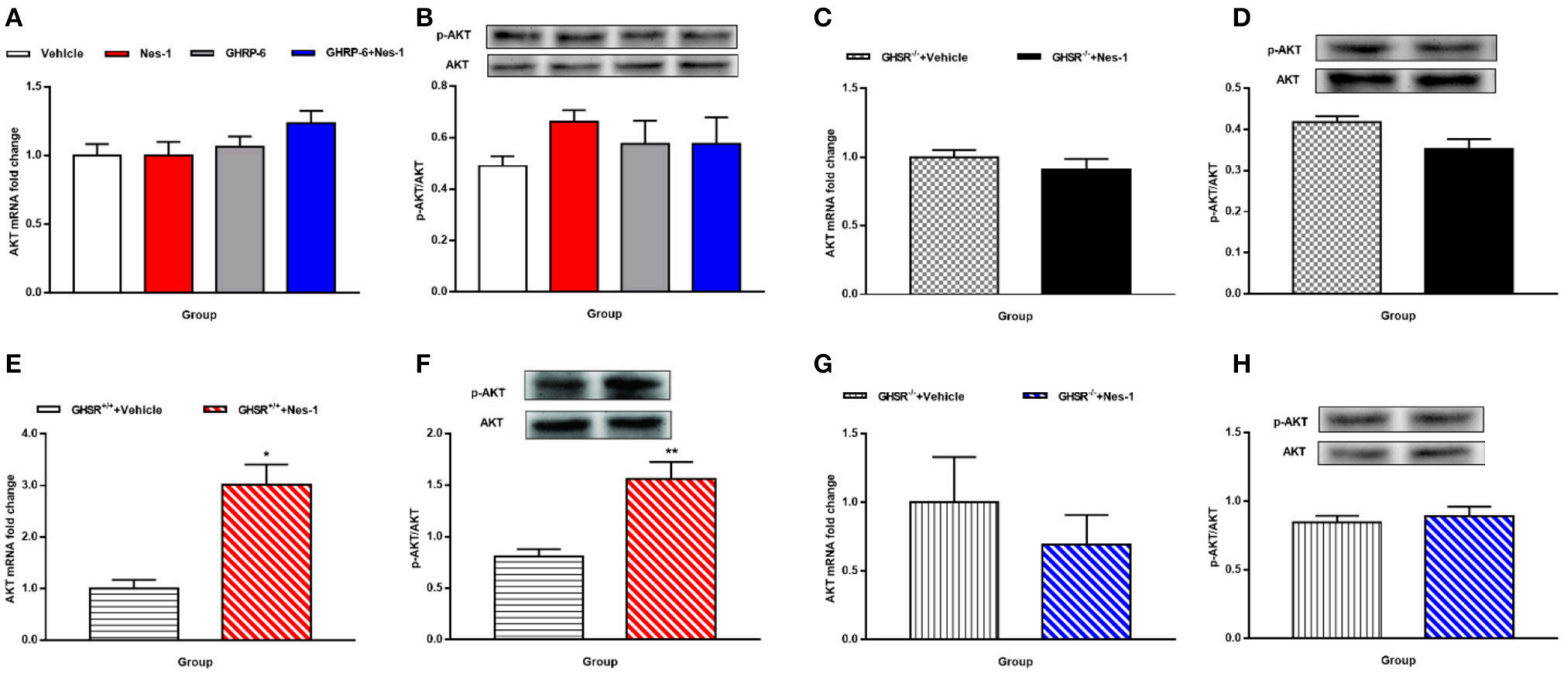
GHSR mediated the effect of nesfatin-1 on AKT phosphorylation in liver of HFD-fed mice. GHSR^+/+^ mice underwent chronic tail vein administration of vehicle, nesfatin-1 (Nes-1), [D-Lys3]-GHRP-6 (GHRP-6) and [D-Lys3]-GHRP-6+nesfatin-1 (GHRP-6+Nes-1) for 12 days. GHSR^−/−^ mice fed underwent chronic tail vein administration of vehicle and nesfatin-1 for 12 days. Insulin (2 IU/kg) was intraperitoneally injected into mice 10 min before mice were sacrificed. **(A,C,E,G)** mRNA expression levels of AKT detected by RT-PCR in liver of **(A)** GHSR^+/+^, **(C)** GHSR^−/−^ mice fed NCD and **(E)** GHSR^+/+^, **(G)** GHSR^−/−^ mice fed HFD. **(B,D,F,H)** P-AKT/AKT level in liver of **(B)** GHSR^+/+^, **(D)** GHSR^−/−^ mice fed NCD and **(F)** GHSR^+/+^, **(H)** GHSR^−/−^ mice fed HFD. **(A,B)** *n* = 5/group; **(C,D)** *n* = 3/group; **(E–H)** *n* = 5/group. Data are expressed as mean ± SEM. ^*^P < 0.05; ^**^P < 0.01 for the effect of nesfatin-1 vs. vehicle. **(A,B)** One-way ANOVA was applied to analyze the statistical difference for multiple groups. **(C–H)** Student's *t*-test was applied to analyze the statistical difference for two groups.

We next investigated the possible molecular mechanisms of glucose-regulating effects of nesfatin-1. First, we examined the phosphorylation levels of AKT as a marker of the insulin signaling pathway. Peripheral IV injection of nesfatin-1 in GHSR^+/+^ mice under NCD increased the mRNA and phosphorylation levels of AKT in skeletal muscle compared with vehicle (Figures 6A,B), but there were no differences in liver (Figures 7A,B). Nesfatin-1's effects in skeletal muscle were all blocked when co-injected with [D-Lys3]-GHRP-6 (Figures 6A,B), or in GHSR^−/−^ mice (Figures 6D,E).

Next, we examined expression levels of the glucose transporter 4 (GLUT4) after injection of nesfatin-1 in skeletal muscle. We found increased mRNA expression levels of GLUT4 in skeletal muscle (Figure 6A), but the result of protein levels was inconclusive (Figure 6C). Similarly, co-injection with [D-Lys3]-GHRP-6 (Figure 6A) and deficiency of GHSR (Figure 6D) inhibited the role of nesfatin-1 in improving the expression of GLUT4 mRNA levels in skeletal muscle. And we did not observe any significant change in GLUT4 protein levels in skeletal muscle of GHSR^−/−^ mice after nesfatin-1 injection (Figure 6F). We further explored GLUT4 membrane translocation by immunofluorescence. Nesfatin-1 elevated GLUT4 translocation (Figures 6G,H,K), and this effect was not seen in GHSR^−/−^ mice (Figures 6I–K). These results suggested that nesfatin-1 modulated blood glucose by increasing membrane translocation, but not protein levels of GLUT4.

Because no significant alterations of AKT were observed in the liver of mice fed NCD (Figures 7A–D), we were eager to further investigate the effects in HFD-mice. Interestingly, unlike the results in NCD-mice, nesfatin-1 increased AKT mRNA (Figure 7E) and p-AKT/AKT (Figure 7F) levels in liver of GHSR^+/+^ mice fed with HFD. The effects of nesfatin-1 observed above was not seen in GHSR^−/−^ mice (Figures 7G,H).

### Nesfatin-1 inhibited c-fos expression induced by fasting in arcuate nucleus

**Figure 8:**
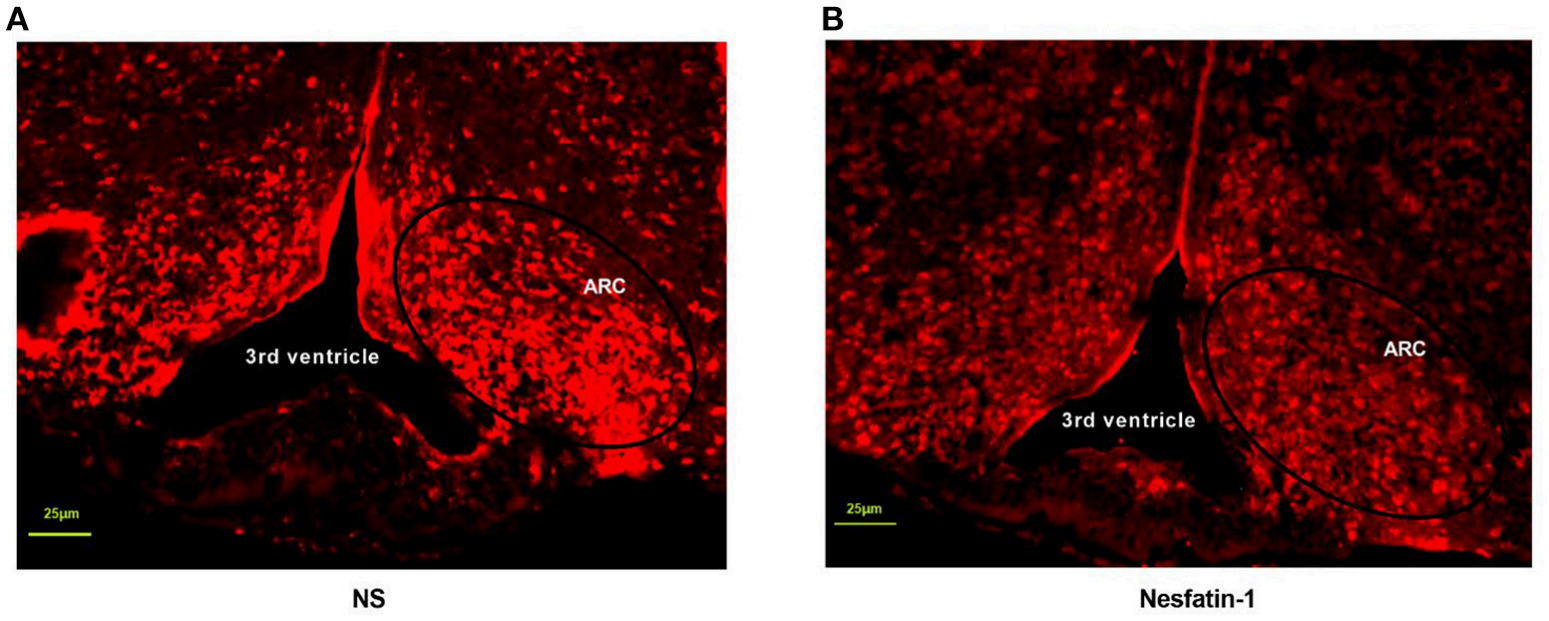
Nesfatin-1 inhibited c-fos expression induced by fasting in arcuate nucleus. GHSR^+/+^ mice were treated with tail vein injection of vehicle and nesfatin-1 after 12 h fasting**. (A)** C-fos expression in hypothalamic ARC of mice injected with vehicle. **(B)** C-fos expression in hypothalamic ARC of mice injected with nesfatin-1. *N* = 3/group.

The next was to investigate the possible mechanisms of interaction between nesfatin-1 and GHSR. We peripherally injected nesfatin-1 in 12 h-fasted mice and measured c-fos expression in ARC by immunofluorescence. It was found that nesfatin-1 markedly suppressed c-fos expression in ARC (Figures 8A,B), suggesting that peripheral nesfatin-1 probably exerted its function at least in part by inhibiting central GHSR signaling.

## Discussion

Although many studies have uncovered the novel function and physiological regulation of nesfatin-1, its receptor has yet to be identified. Nesfatin-1 was found interacting with a G protein-coupled receptor in the rat brain (3), and its peripheral and central localization was described by ^125^I-nesfatin-1 autoradiography in 2016 (40). A recent article suggested that nesfatin-1 might have anti-inflammatory effects acting through GHSR (41), confirming our findings regarding the relationship between nesfatin-1 and GHSR. Based on the previous findings in this field, we investigated the relationship between GHSR and nesfatin-1. Here we first raised a novel hypothesis that nesfatin-1 regulated satiety and glucose metabolism partly through GHSR, and then verified it experimentally.

In our study, mice were observed to have elevated blood glucose and glucose intolerance during OGTT after 8 weeks feeding of HFD (Figure 1F), which is consistent with previous studies showing impaired glucose homeostasis in the early stage of HFD (42, 43). And thus, our HFD model is sufficient to be studied in abnormal glucose metabolism. When we monitored blood glucose of wild-type and GHSR^−/−^ mice before administration, we found an interesting phenomenon: the blood glucose difference between the two types of mice was inconsistent under different energy states (Figures 1B,E). A previous study demonstrated that ghrelin played an important role during fasting by causing the body to switch from glucose utilization to fatty acid oxidation, thus conserving glucose for vital organ functions (44). We thus presumed that the decreased blood glucose caused by fasting triggered ghrelin functioning by promoting this activity via its receptor.

We also investigated plasma levels of nesfatin-1 in the two mice models. A previous study showed significantly reduced circulating nesfatin-1 levels in rats fasted for 24 h (45). Li et al. confirmed this finding in the stomach of mice (fasted for 24 h) (46). By contrast to these studies, in our study, mice under *ad libitum* and fasting conditions (12 h) showed no difference in plasma nesfatin-1 levels. The inconsistency may relate to the time of fasting or the different strains. To our surprise, the plasma levels of nesfatin-1 increased significantly after fasting for 12 h in GHSR^−/−^ mice, suggesting that GHSR may involve in the reaction of nesfatin-1 secretion in various energy states, or the increased circulating nesfatin-1 was due to loss of function sites (ghrelin receptor) in liver or skeletal muscles, thus become one of the evidence supporting our hypothesis that the glycemic effect of nesfatin-1 requires GHSR. Nevertheless, the concrete mechanism remains uninvestigated.

Previous studies showed conflicting data regarding the peripheral effects of nesfatin-1 on food intake. Shimizu et al. found that intraperitoneal injection of nesfatin-1 displayed an anorexigenic role in mice, but this occurred only when the dose was >1,000-fold higher than the dose required by central administration (12). Consistent with what we found, Geobel et al. observed no reduced dark phase food intake in wild-type mice fed NCD induced by peripheral injection of nesfatin-1 (11). The dose of the former (12) was about 75-fold higher than what we used, and the effect of peripheral nesfatin-1 on satiety proved to be dose-independent (12). Thus, we suspect that the dose of nesfatin-1 injected peripherally to mice contributes much to the contradictory results. In addition, in GHSR^+/+^ mice fed with HFD, intravenous injection of nesfatin-1 reduced dark phase ingestion compared with the control group, a finding similar to what Shimizu et al. found (12). Our observation in mice fed with different diets implies that mice with metabolic disorders were more sensitive to the anorexigenic effect of nesfatin-1 than were mice in normal energy metabolism states at the same dose of nesfatin-1. More importantly, the effects of nesfatin-1 on food intake were attenuated by [D-Lys3]-GHRP-6 in HFD mice, and we confirmed these findings in GHSR^−/−^ mice. In our previous study, nesfatin-1 stimulated fatty acid oxidation in type 2 diabetes-induced mice (37). To the best of our knowledge, oxidation of fatty acids has always been closely related to regulation of food intake (47). Therefore, there is a possibility that nesfatin-1 exerts its anorexic role in HFD-mice by regulating lipid metabolism that also requires the participation of GHSR.

There were also conflicting results regarding the effects of nesfatin-1 on glucose metabolism. Early investigations showed the positive effects of nesfatin-1 on insulin (31, 48). Consistent with our results, some investigators found that peripheral infusion of nesfatin-1 showed a glucose-reducing effect (13, 14). On account of the difference in experimental conditions, Gonzalez et al. found that during the OGTT, blood glucose was similar in nesfatin-1 and saline-treated rats (14). Another study suggested that intravenous injection of nesfatin-1 did not affect blood glucose in euglycemic mice (49). Of greater significance from the clinical perspective was that decreased glucose blood glucose was observed in GHSR^+/+^ mice fed with HFD after long-term injection of nesfatin-1, suggesting potential treatment effects of chronic treatment with nesfatin-1 in patients suffering from related metabolic diseases. Just as we assumed previously, we supposed that the chronic effects of nesfatin-1 on decreasing blood glucose observed in HFD-mice were also related to lipid metabolism. This relationship requires further exploration. Though some conflicts exist, what is more important is that we found that the deficiency of GHSR also completely blocked the glucose-regulating effects of nesfatin-1, further supporting our conjecture.

In addition to these results, we further investigated whether GHSR was involved in the downstream molecular pathway of glucose metabolism regulated by nesfatin-1. A study suggested that AKT was critical for stimulating glucose uptake by skeletal muscle (50), and that AKT phosphorylation induced by insulin resulted in membrane translocation of GLUT4 (51). More decisively, a recent study demonstrated that continuous peripheral administration of nesfatin-1 elevated the activation of AKT as well as GLUT4 expression in an insulin-dependent manner in specific peripheral tissues (13). Therefore, we selected AKT and GLUT4 as the indicators to examine. In agreement with the up-regulated level of p-AKT/AKT in skeletal muscle induced by continuous subcutaneous administration of nesfatin-1 found by Li et al. (13), we also found that IV injection of nesfatin-1 increased mRNA and phosphorylation levels of AKT in skeletal muscle. However, Li et al. found that peripheral nesfatin-1 failed to increase hepatic levels of p-AKT/AKT in HFD-fed mice, only augmenting it in NCD-fed mice, exactly the opposite of our finding. However, Yang et al. found that central nesfatin-1 enhanced hepatic levels of p-AKT/AKT in rats fed HFD, supporting our findings (49) that no significantly altered p-AKT/AKT levels were observed in the livers of mice fed NCD. We conjectured that the response of nesfatin-1 on regulating glucose metabolism mediated by AKT in liver only occurred in situations of metabolic disturbance. Yang et al. also observed markedly improved glucose uptake levels caused by central nesfatin-1 in skeletal muscle (49). The alteration of levels of GLUT4 mRNA and membrane translocation in muscle was congruent with the findings of the abovementioned studies. However, no significant difference as discovered on Western blot between groups with disparate treatments. We attribute this contradiction to the different site of activity (mRNA or protein) modulated by peripheral nesfatin-1, or nesfatin-1 may regulate blood glucose by increasing membrane translocation but not protein levels of GLUT4. We also confirmed previous observations as to the specific-tissue effect of nesfatin-1 on glucose metabolism (13, 49). Most of all, both AKT and GLUT4 were similar in GHSR^−/−^ mice regardless of treatment with nesfatin-1 or vehicle, suggesting that GHSR played a crucial role in AKT/GLUT4 passage activated by peripheral injection of nesfatin-1.Our previous hypothesis was that the partial role of nesfatin-1 in satiety and metabolism was indirect mediated by ghrelin secretion, or direct by modulating GHSR activity. Kerbel et al. showed that central administration of nesfatin-1 diminished the expression of ghrelin in goldfish (26). However, we found no alteration of ghrelin levels in GHSR^+/+^ mice after peripheral injection of nesfatin-1 (data not shown). Thus, peripheral nesfatin-1 most likely exerts part of its effect directly through GHSR signaling. GHSR, as a G protein-coupled receptor, was found to function in a highly ligand-independent way, highlighting the potential of GHSR inverse agonists in the field of obesity therapy (25). Nesfatin-1 has almost completely opposite effects to those of ghrelin in terms of energy metabolism, while its function is the same as the identified GHSR inverse agonists (25, 52). In addition, peripheral GHSR inverse agonist inhibited fasting-induced c-fos expression in the hypothalamic ARC (52), and we demonstrated that peripheral nesfatin-1 exerted the identical effect on central c-fos expression as an GHSR inverse agonist. Therefore, it is reasonable to regard nesfatin-1 as an endogenous inverse agonist of the GHSR. Hence, it is possible that nesfatin-1 has an effect on influencing the structure or activity of GHSR. This hypothesis requires verification through further studies.

Here we expanded the existing study regarding the role of peripheral nesfatin-1 on feeding behavior and glucose metabolism. The model of HFD-mice is sufficient for providing potential therapeutic methods in patients with metabolic diseases, especially in patients with glucose metabolic disorders, such as type 2 diabetes mellitus. To the best of our knowledge, no analogical study regarding the relationship between nesfatin-1 and GHSR has been published previously. Previous findings implied some correlation between nesfatin-1 and GHSR, and here we confirmed this directly by using GHSR antagonists as well as GHSR^−/−^ mice. Because the receptor for nesfatin-1 remains unknown, our study develops a new direction for the investigation of nesfatin-1 receptor. In light of the potential clinical application value in maintenance of glucose metabolic disorders revealed by nesfatin-1, the elucidation of the receptor appears to be particularly consequential. More importantly, GHSR signaling is also an attractive target for the therapy of obesity-related metabolic disease; however a study showed that its ligand, ghrelin, was lower in obese subjects than in a normal group (53). Hence, this limits the effects of the exploration on blockage of ghrelin including inactivation and the use of GHSR antagonists in treatment of metabolic disorders. Therefore, GHSR inverse agonists exhibit unique advantages. Nevertheless, there are remain some disadvantages. The present study was limited to *in vivo* experiments. There was no exploration of molecular biology, preventing us from directly observing the action of nesfatin-1 on GHSR. Our future studies will compensate for these limitations. According to our results, the application of other metabolic disorder-associated experimental models (e.g., type 1 diabetes and type 2 diabetes) may further advance the relationship between the nesfatin-1 and GHSR.

In conclusion, the main finding of the present study was that GHSR was required for the role of IV administration of nesfatin-1 on food intake and glucose metabolism in mice fed both NCD and HFD. GHSR was involved in the AKT/GLUT4 pathway modulated by peripheral nesfatin-1.

## Author contributions

X-TF and ZT designed the experiments, performed the experiments, researched data, and wrote the manuscript. S-ZL and TZ designed the experiments, performed the experiments, and researched data. RW, C-SZ, L-XW, and J-HY performed the experiments. J-LL and YZ contributed to discussion of the experiments. JD directed the project, contributed to discussion, reviewed and edited the manuscript. JD as the corresponding author had full access to all the data in the study and had final responsibility for the decision to submit for publication. X-TF, ZT, S-ZL, and TZ contributed equally to this work.

### Conflict of interest statement

The authors declare that the research was conducted in the absence of any commercial or financial relationships that could be construed as a potential conflict of interest.
